# Relationship between Interleukin-6 Gene Polymorphism and Hippocampal Volume in Antipsychotic-Naïve Schizophrenia: Evidence for Differential Susceptibility?

**DOI:** 10.1371/journal.pone.0096021

**Published:** 2014-05-02

**Authors:** Sunil Vasu Kalmady, Ganesan Venkatasubramanian, Venkataram Shivakumar, S. Gautham, Aditi Subramaniam, Dania Alphonse Jose, Arindam Maitra, Vasanthapuram Ravi, Bangalore N. Gangadhar

**Affiliations:** 1 The Schizophrenia Clinic, Department of Psychiatry, National Institute of Mental Health and Neuro Sciences, Bangalore, India; 2 Translational Psychiatry Laboratory, Cognitive Neurobiology Division, Neurobiology Research Centre, National Institute of Mental Health and Neuro Sciences, Bangalore, India; 3 National Institute of Biomedical Genomics, Kalyani, India; 4 Department of Neurovirology, National Institute of Mental Health and Neuro Sciences, Bangalore, India; Wayne State University, United States of America

## Abstract

**Background:**

Various lines of evidence including epidemiological, genetic and foetal pathogenetic models suggest a compelling role for Interleukin-6 (IL-6) in the pathogenesis of schizophrenia. IL-6 mediated inflammatory response triggered by maternal infection or stress induces disruption of prenatal hippocampal development which might contribute towards psychopathology during adulthood. There is a substantial lack of knowledge on how genetic predisposition to elevated IL-6 expression effects hippocampal structure in schizophrenia patients. In this first-time study, we evaluated the relationship between functional polymorphism *rs1800795* of *IL-6* and hippocampal gray matter volume in antipsychotic-naïve schizophrenia patients in comparison with healthy controls.

**Methodology:**

We examined antipsychotic-naïve schizophrenia patients [N = 28] in comparison with healthy controls [N = 37] group matched on age, sex and handedness. Using 3 Tesla – MRI, bilateral hippocampi were manually segmented by blinded raters with good inter-rater reliability using a valid method. Additionally, Voxel-based Morphometry (VBM) analysis was performed using hippocampal mask. The IL-6 level was measured in blood plasma using ELISA technique. SNP *rs1800795* was genotyped using PCR and DNA sequencing. Psychotic symptoms were assessed using Scale for Assessment of Positive Symptoms and Scale for Assessment of Negative Symptoms.

**Results:**

Schizophrenia patients had significantly deficient left and right hippocampal volumes after controlling for the potential confounding effects of age, sex and total brain volume. Plasma IL-6 levels were significantly higher in patients than controls. There was a significant diagnosis by *rs1800795* genotype interaction involving both right and left hippocampal volumes. Interestingly, this effect was significant only in men but not in women.

**Conclusion:**

Our first time observations suggest a significant relationship between *IL-6 rs1800795* and reduced hippocampal volume in antipsychotic-naïve schizophrenia. Moreover, this relationship was antithetical in healthy controls and this effect was observed in men but not in women. Together, these observations support a “differential susceptibility” effect of *rs1800795* in schizophrenia pathogenesis mediated through hippocampal volume deficit that is of possible neurodevelopmental origin.

## Background

Neurodevelopmental model postulates schizophrenia as a behavioural outcome of aberration in neurodevelopmental processes that begins long before the onset of clinical symptoms and is caused by a combination of genetic and environmental factors [1]. Among the various parameters that comprise gene-environment interactions, the crosstalk between genetic factors and obstetric complications has been put forth as one of the important mechanisms that increase the risk towards schizophrenia [2]. In this context, it is noteworthy that prenatal maternal infections with resultant persistent pro-inflammatory state leading to aberrant neurodevelopment are among the important obstetric complications that have been shown to confer increased risk for schizophrenia[3].

Epidemiological observations strongly support the association between elevated risk for schizophrenia in the offspring and prenatal exposure to influenza, toxoplasma, rubella, genital-reproductive infections & various other infections [4]. Research studies involving animal models examining the resultant maternal immune activation (MIA) secondary to prenatal infection has offered robust support to these epidemiologic findings through replicated observations that MIA causes phenotypes analogous to those found in patients with schizophrenia[5]. In this context, it is noteworthy that Interleukin-6 (IL-6) – a predominantly pro-inflammatory cytokine – has been shown to play a pivotal role in mediating the aberrant influence of MIA on fetal brain development leading to putative abnormalities that might possibly underlie the pathogenesis of schizophrenia [6].

In addition, various lines of evidence suggest that there is a compelling role for IL-6 to underlie the pathogenesis of schizophrenia as summarized by the following observations: i) rigorous meta-analytic studies supporting significantly higher serum levels of IL-6 in schizophrenia patients that correlate with symptom severity [7]; ii) association of schizophrenia with *IL-6* gene polymorphism [8], [9]; iii) potential influence on hippocampus by peripheral IL-6 with hippocampus being the one of the most important brain regions implicated in schizophrenia[10]; iv) IL-6 playing a vital role in established models like ‘ketamine model’ of schizophrenia [11]; v) IL-6 being implicated in foetal-pathogenetic model of neurodevelopmental aberrations in schizophrenia [6].

The human *IL-6* gene is located on chromosome 7p15–7p21 and regulation of *IL-6* expression is influenced by subtle variations in the promoter. A functional G→C single-nucleotide polymorphism at position -174 (*rs1800795*) of the promoter has been described. Relationship between *rs1800795* genotype and plasma levels of IL-6 is complex because of its involvement in gene × environment interaction [12]. The *rs1800795* transversion is shown to affect IL-6 gene expression by modulating binding of transcription factors such as GATA1 [13], with few studies showing that the GG allele is associated with greater induction of IL-6 compared with the GC or CC alleles [14]. This modulation, however, may be subjective to presence of environmental conditions that induce the transcription factor activity [12]. Because of the necessary precondition, phenotypic manifestation of *rs1800795* is not universal. For instance, impact of *rs1800795* (in terms of association with increased IL-6 levels) has been found in inflammation-related conditions such as ageing [15], systemic-onset juvenile chronic arthritis [14], liver cirrhosis, hepatocellular carcinoma [16], primary Sjogren's syndrome [17] as well as in medicated schizophrenia patients [8]. However, lack of this association [18] or even reversal of such association [19] has been reported in healthy individuals, which possibly reflects absence of environmental triggers of transcriptional activity. A recent meta-analysis provides evidence that *rs1800795* polymorphism is not significantly associated with circulating IL-6 levels in a normal population [20].

Interestingly, the regulation of IL-6 production by this polymorphism is more pronounced in neonates than in adults [21], indicating that *rs1800795* might play a prominent role in determining the inflammatory response during the phases of early environmental adversities in pathogenesis of schizophrenia. Hence, it is possible that maternal immune activation during pregnancy (which is more prevalent in schizophrenia patients compared to healthy controls [22], [23]) might result in hyper-inflammatory IL-6 response in individuals with GG allele. Further, *rs1800795* can moderate the impact of alterations in functioning of HPA axis and sympathetic nervous system which might result from neonatal immune challenge, such that C allele carriers would be protected from the heightened inflammation related to adverse environmental conditions [12]. Though these background works suggest a critical role for IL-6 in the pathogenesis of schizophrenia, one has to acknowledge this is clearly a candidate gene approach since currently there is only a limited support for the proposed SNP in terms of evidence from genome wide association studies on schizophrenia pathophysiology.

In relation to IL-6 gene polymorphisms, various lines of evidence support a significant relationship between *rs1800795* and pre-term birth - another important obstetric complication that has been associated with elevated risk for schizophrenia [24], [25]. Moreover, prematurity at birth has been consistently shown to be linked with hippocampal volume deficit in a recent meta-analysis [26]; in addition, it has been demonstrated such hippocampal volume aberrations in preterm infant might have long lasting adverse impact on cognitive functions [27]. These observations become significant with relevance to schizophrenia, since hippocampus is a critical brain region implicated in the pathogenesis of this disorder [28], [29], [30], [31]; more specifically, hippocampal volume deficit in schizophrenia has been shown to be significantly influenced by obstetric complications [32].

Relationship between peripheral IL-6 and hippocampal volume has been reported by previous studies. One study that has examined middle-aged, healthy individuals demonstrated a significant inverse relationship between the plasma IL-6 level and left hippocampal gray matter volume [10]. Another study has shown that in patients with first-episode psychosis, IL-6 expression in the peripheral leucocyte significantly predicted a smaller left hippocampal volume [33].

Thus, several lines of evidence as reviewed above implicate a compelling role for elevated IL-6 in the pathogenesis of schizophrenia possibly through disruption of prenatal hippocampal development. Increased level of IL-6 has been associated with smaller hippocampal volume. In the context of IL-6 gene promoter polymorphism, since GG allele is associated with increased expression of IL-6 gene in the presence of environmental conditions, one would predict this allele to be associated with smaller volume of hippocampus in schizophrenia patients. However, to the best of our knowledge, the effect of *IL-6* promoter polymorphism on hippocampal volume is yet to be evaluated in schizophrenia. In this study, we evaluated the relationship between *IL-6* promoter polymorphism and hippocampal gray matter volume in antipsychotic-naïve schizophrenia patients (N = 28) in comparison with healthy controls (N = 37). We hypothesized that schizophrenia patients with GG allele will demonstrate significantly deficient hippocampus volume in comparison with healthy controls.

## Methods

Patients attending the clinical services of the National Institute of Mental Health & Neurosciences (India), who fulfilled DSM-IV criteria for schizophrenia and were never treated with any psychotropic medications including antipsychotics & not having substance abuse [n = 28; age = 29.9±5.7; 14-males], were examined in this study. The diagnosis of schizophrenia was established using Mini International Neuropsychiatric Interview Plus [34], which was confirmed by another psychiatrist through an independent clinical interview. The details related to illness onset and antipsychotic-naïve status were carefully ascertained by reliable information obtained from at least one reliable adult relative. Psychotic symptoms were assessed using Scale for Assessment of Positive Symptoms (SAPS) and Scale for Assessment of Negative Symptoms (SANS). Healthy controls (N = 37) (age = 27.4±5.6 years; 20-males), who volunteered for study, were screened to rule out any psychiatric diagnosis using the MINI as well as a comprehensive mental status examination. None of the controls had family history of psychiatric disorder in first-degree relatives. To avoid the potential confounding effect of differential handedness, only right handed subjects were included in this study.

Patients and controls did not have features suggestive of alcohol abuse/dependence. None used stimulant or opiate drug. None had history or clinical feature suggestive of neurological/medical disorder. None had abnormal movements as assessed by Abnormal Involuntary Movements Scale. Clinical assessments and blood sample collection were performed on the same day before starting antipsychotics. After complete description of study to the subjects, written informed consent was obtained. The research protocol was reviewed and approved by the National Institute of Mental Health and Neurosciences (NIMHANS) ethics committee.

### MRI Acquisition

MRI was done with 3.0 Tesla scanner (Achieva, Philips, Best, The Netherlands). T1 weighted images were acquired using the following parameters: TR = 8.1 msec, TE = 3.7 msec, nutation angle = 8 degree, FOV = 256 mm, slice thickness = 1 mm without inter-slice gap, NEX = 1, matrix  = 256×256. The images were transferred on to a personal computer (PC) platform. They were stored with coded identification.

### Hippocampus Volumetry

**Figure 1:**
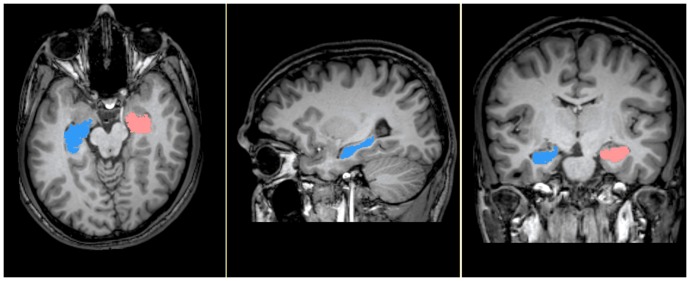
Cerebral MRI depicting manual segmentation of hippocampus. Figure shows MRI with segmented hippocampus in axial, sagittal & coronal sections within 3D-Slicer software interface.

Bilateral hippocampi were measured using MRI scans with the software ‘3D Slicer 3.4’ (http://www.slicer.org/). The structure was outlined by the rater using the computer mouse controlled pointer and measured using semi-automated three-dimensional interactive method (Figure-1). The gray matter area was marked in each consecutive coronal slice along the anterior to posterior using anatomical landmarks validated using review of literature [35]. Anteriorly, first appearance of alveus was identified to reliably differentiate hippocampus from amygdala [36].Superior border of hippocampus is covered by the alveus and adjoins to cerebrospinal fluid while the inferior border was identified by the white matter of the parahippocampal gyrus below the subiculum. The lateral ventricle CSF was used as an external landmark to define the lateral extent. Medial border was defined superiorly by the CSF of the cisterna ambiens and inferiorly by vertical arbitrary line placed at the dorsomedial tip of the white matter of the parahippocampal gyrus[37]. Coronal slice where an ovoid gray matter starts to appear inferiomedially to the trigone of the lateral ventricle marked the posterior end [38]. Sagittal and axial sections were used to confirm the anatomical landmarks. Further, after tracing on coronal section, imperfections were rectified on sagittal plane, such as excluding any areas above hippocampal body (fimbria) and confirming the posterior extent of the hippocampal tail. The total brain volume, which was used as a covariate in statistical analyses to control for the potential confounding effect of the global brain size, was automatically computed using established methods (FMRIB Software Library (FSL); www.fmrib.ox.ac.uk/fsl/). In order to assess reliability of the measures, another trained rater blind to diagnosis of subject, independently carried out hippocampal volumetry in a random subset of data (n = 10). Excellent reliability was ascertained with intra-class correlation coefficient of more than 0.95.

### Voxel Based Morphometry [VBM]

T1-weighted images were processed using SPM8 (Wellcome Trust Centre for Neuroimaging, UCL, London; UK; http://www.fil.ion.ucl.ac.uk/spm) implemented in the VBM Toolbox 8 (http://dbm.neuro.uni-jena.de/vbm.html) under MatLab 7.8.0 (The MathWorks Inc., Sherborn, MA, USA). Standard routines and default parameters of the VBM 8 toolbox were applied. After setting the image origin to the anterior commissure, images were bias corrected, pre-registered to standardized International consortium for brain mapping (ICBM- East Asian brains) space using affine transformation with regularization and segmented using the “unified segmentation” approach [39]. SPM8 segmentation was based on a modified gaussian mixture model to avoid misclassification. Spatial adaptive non local means (SANLM) filter was applied to remove noise while preserving edges. To remove isolated voxels of one tissue class within a cluster of voxels belonging to a different tissue class, a hidden Markov random field model weighting was used. Spatial normalization was performed with high-dimensional Diffeomorphic Anatomical Registration using the Exponentiated Lie algebra (DARTEL) template [40]. Grey matter segments were modulated by the Jacobian determinants of the deformations to account for local expansion and compression introduced by non-linear transformation. Finally, the gray matter images were smoothed with an 8-mm full-width at half-maximum (FWHM) isotropic gaussian kernel. This was done to reduce errors related to inter-subject variability in local anatomy and to render the imaging data more normally distributed. Bilateral hippocampal mask was created using the Wake Forest University school of medicine (WFU) Pickatlas [41].

Total brain tissue volume was estimated using Sienax tool of FSL package [42]. Statistical parametric maps were examined for effect of diagnosis as well as diagnosis by genotype interaction of voxel-wise hippocampal gray matter volume using ANCOVA model controlling for confounding effects of sex, age and total intracranial volume. In order to avoid possible edge effects between different tissue types, all voxels with gray matter values of less than 0.1 (absolute threshold masking) were excluded. Further, threshold of uncorrected p<0.05 was applied, since GLM analyses were limited to a priori region contained within the hippocampal mask.

### IL-6 Assay

Blood samples were collected between 0800 and 0900 hrs. (A.M) after 12-hour overnight fast from ante-cubital vein into K_2_ EDTA vacutainer tubes (Becton & Dickinson, U.S.A). Plasma were collected within 30 minutes of collection by centrifugation for15 minutes at 1000 x g, which were then aliquoted and stored at −80°C. Quantitative sandwich enzyme immunoassay for IL-6 was done using commercial ELISA method with sensitivity <0.70 pg/mL (R&D Systems, MN, USA) following manufacturer's instructions. All samples were coded; thawed only once and analyzed by the same investigator, who was blind to the clinical situation.

### SNP Genotyping

DNA extraction was carried out using commercial spin column method (Qiagen, Inc.). First, the samples were treated with protease and then with WBC lysis buffer by incubating the mixture at 56 C for 10 min. Later, digested samples were treated with ethanol and then, subjected to spin column for isolation of genomic DNA. The columns were washed twice and then DNA is eluted in elution buffer. Extracted DNA was checked on agarose gel and stored at -80 C for later use.

The PCR amplification of the *IL-6* promoter was performed with published primers [43] using True Allele PCR Mix protocol in Veriti Thermal Cycler (Applied Biosystems). The PCR products were checked on agarose gel, purified using silica spin column (Invitrogen) and sequenced using Big Dye Terminator v3.1. cycle sequencing kit (Applied Biosystems). Sequencing was carried out on 3730XL Genetic Analyzer (Applied Biosystems) with PCR primers as well as two additional internal primers published previously [44]. SeqScape v2.7 software (Applied Biosystems) was used for the assembly of the sequence data. The reference sequence of the *IL-6* promoter region used for analysis was downloaded from the Ensembl Genome Browser release 67 - GRCh37, May 2012 [45].

All subjects were of Indian origin. Expected minor (C) allele frequency of *rs1800795* in Indian population is 0.12 [GIH population - HapMap database Release 27]. (http://hapmap.ncbi.nlm.nih.gov/cgi-perl/gbrowse/hapmap27_B36/?name=SNP%3Ars1800795) [46]. Due to the low prevalence of the *rs1800795* C allele in our population, only 2 CC homozygotes out of total 65 subjects were detected. Hence, the genotypes are grouped into two as “GG” and “GC/CC” using the dominant model, similar to some of the earlier *rs1800795* studies[47]. Data were tested for normality using the Shapiro–Wilks test. Data analysis was performed using the SPSS-11.0 using the following statistics: student's t-test (two-tailed), chi-square test, Pearson's correlation, and general linear model based multivariate & univariate analysis of covariance (ANCOVA).

## Results

The age and sex ratio in both patient and control groups was similar. The genotypes were consistent with Hardy-Weinberg equilibrium proportions in both the groups [Patient: χ2 = 0.6; p = 0.4; Control: χ2 = 1.5; p = 0.2]. Distribution of the minor allele and genotypes for *rs1800795* polymorphism are presented in Table-1. There was no significant difference in the frequency distribution of *rs1800795* alleles and genotypes.

**Table 1:** Comparative Profile of *rs1800795* genotype distribution between patients & controls.

rs1800795	Frequency [number of samples]		?2	p
	Patients [28]	Controls [37]		
Minor Allele (C)	0.13 [7]	0.16 [12]	0.12	0.73
CC	0 [0]	0.05 [2]		
GC	0.25 [7]	0.22 [8]	1.60	0.45
GG	0.75 [21]	0.73 [27]		

Schizophrenia patients had significantly deficient left and right hippocampal volumes after controlling for the potential confounding effects of age, sex and total brain volume (Table- 2) Also, plasma IL-6 levels were significantly higher in patients than controls. However, the levels did not differ between GG and GC/CC genotypes [Patient: t = 0.3; p = 0.9; Control: t = 0.2; p = 0.8].

**Table 2:** Comparative profile of schizophrenia patients and healthy controls.

Characteristic	Patients	Controls	Statistic	p
N	28	37		
Age [Years] *	29.9±5.7	27.4±5.6	t = 1.8	0.1
Sex Ratio [M:F] $	14∶14	20∶17	?^2^ = 0.1	0.7
Left Hippocampal Volume [mL]^#^	2.4±0.4	2.7±0.3	F = 9.8	0.003
Right Hippocampal Volume [mL]^#^	2.6±0.5	2.9±0.3	F = 10.5	0.002
Plasma IL-6 [pg/mL] * ^,£^	2.2±1.8	1.4±0.8	t = 2.3	0.03
*IL-6* Genotype [GG: GC/CC]$	21∶7	27∶10	?^2^ = 0.03	0.9

* - Independent samples t-test;

$ - Chi-Square test; ^#^ - ANCOVA.

£ - data was available for 25 patients and 33 controls.

**Figure 2:**
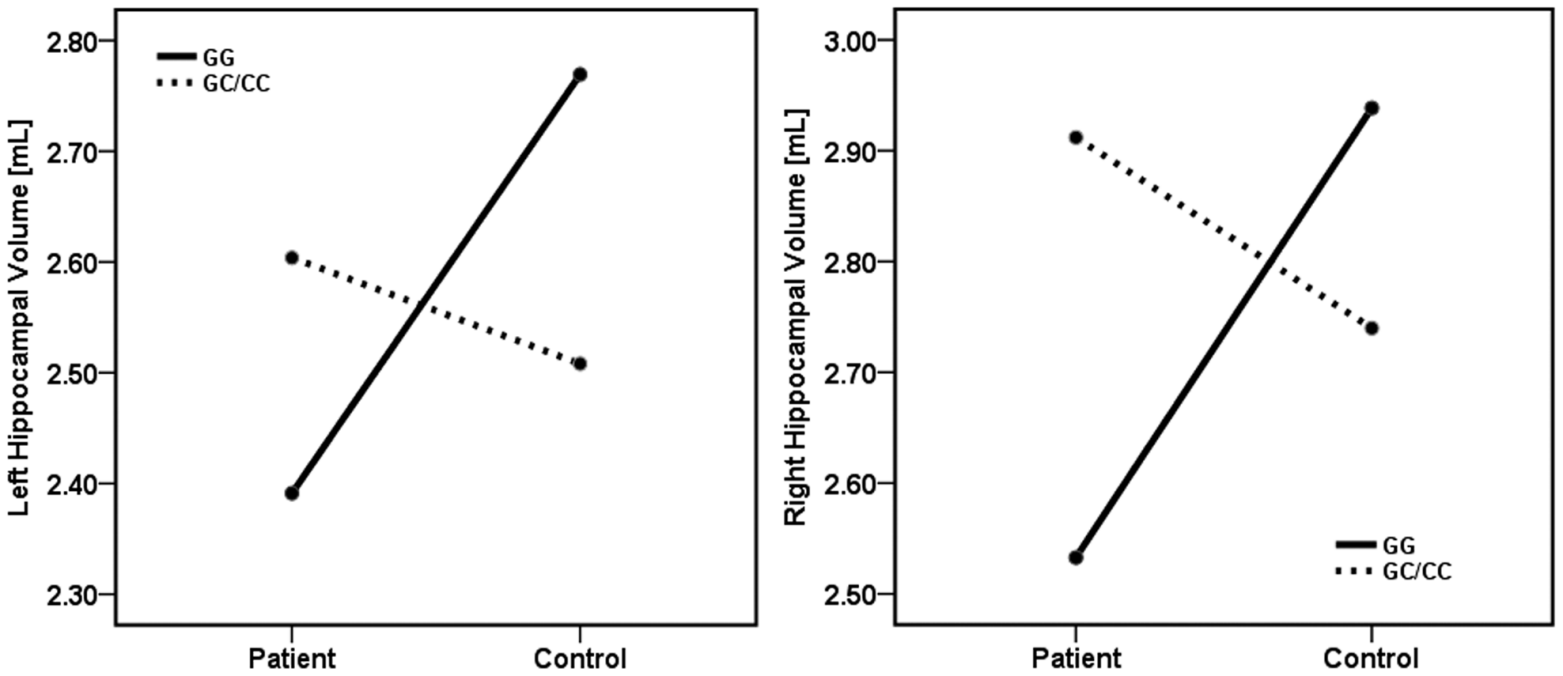
Significant diagnosis-by-genotype interaction on hippocampal volumes. Figure shows significant diagnosis by genotype interaction involving both right and left hippocampal volumes – i.e. the effect of *rs1800795* genotypes [GG & GG/GC] on hippocampal volume was found to be antithetical between patients and controls.

**Figure 3:**
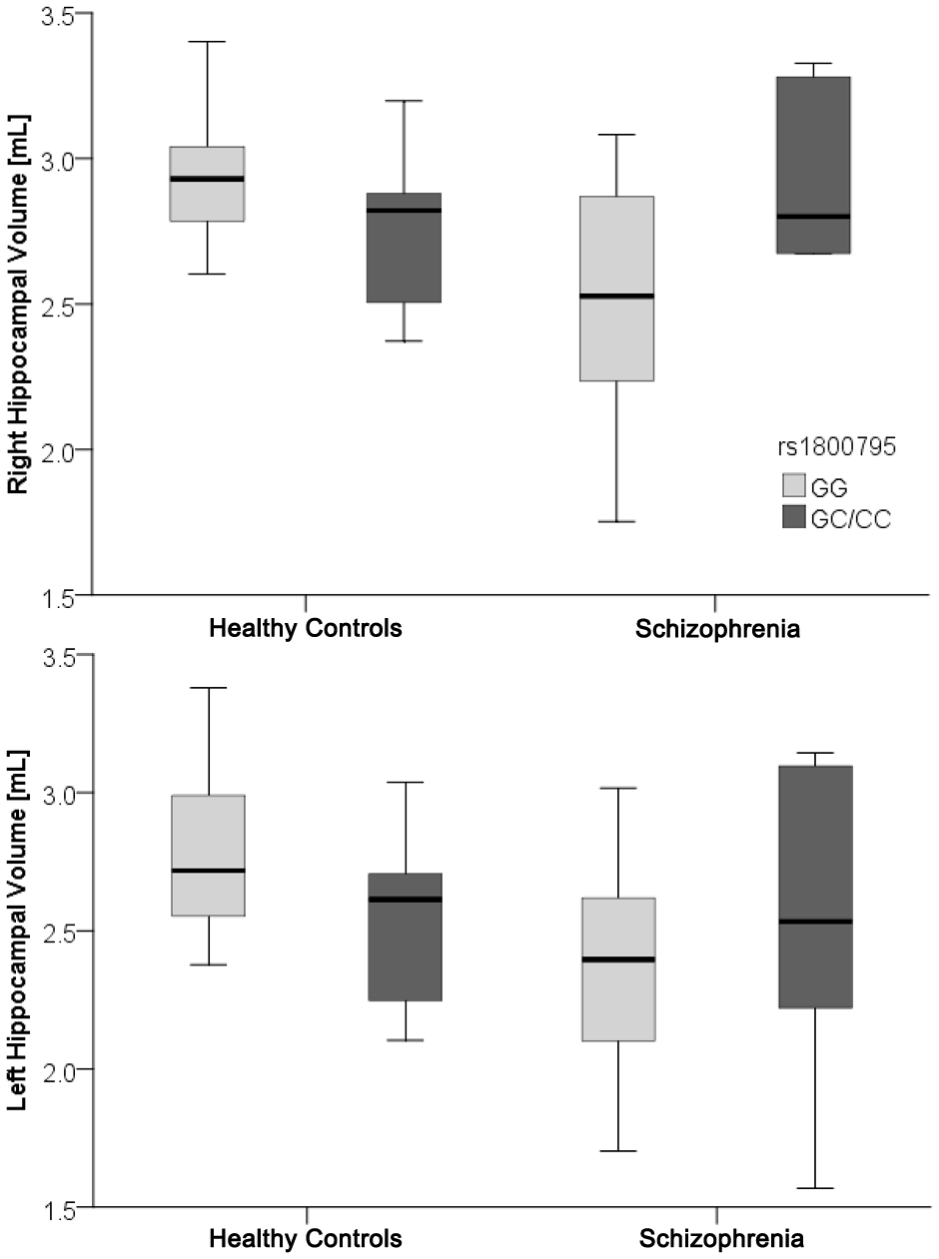
Box plot depicting significant diagnosis-by-genotype interaction on hippocampus volume between patients and controls. Figure shows box plot depicting significant diagnosis by genotype interaction involving both right and left hippocampal volumes – i.e. the effect of *rs1800795* genotypes [GG & GG/GC] on hippocampal volume was found to be antithetical between patients and controls; the interaction boxplot shows the five statistics of hippocampal volume (minimum, first quartile, median, third quartile, and maximum) for each genotype per group.

Multivariate ANCOVA on bilateral hippocampal volume, controlling for the potential confounding effects of age, sex and total brain volume revealed a significant ‘diagnosis by genotype’ interaction [F = 5.0; p = 0.01] involving both right [F = 10.2; p = 0.002] and left [F = 6.6; p = 0.013] hippocampal volumes (Figure-2 & Figure-3). Follow-up analyses to uncover the effect of genotype identified that in the subgroup of subjects who carried “GG” genotype, the hippocampal volumes were significantly deficient in patients in comparison with healthy controls [Right: F = 23.2; p<0.001; Left: F = 20.5; p<0.001], whereas in the subgroup with “GC/CC” genotype, the hippocampal volumes did not differ significantly [Right: F = 0.7; p = 0.4; Left: F = 0.7; p = 0.4]. Within healthy controls, GG homozygotes had significantly larger bilateral hippocampal volume compared to GC/CC carriers [Right: F = 5.2; p = 0.03; Left: F = 4.6; p = 0.04]; on the contrary, the reverse was true in patients [but significant only for right side; Right: F = 5.5; p = 0.03; Left: F = 1.7; p = 0.2]. [ As the control group consisted of a larger number of subjects than the patients' group, it is possible that this might have differentially influenced the variance of the measurements between groups. To explore this further, we performed additional analyses involving patients (N = 28) and equal number of controls (N = 28) with one-to-one matching. The results of these analyses did not differ from the observations from the whole group (supplementary material - S1)].

Further, exploratory analyses of ‘diagnosis by genotype by sex’ interaction was found to be significant for left [F = 5.6; p = 0.02] but only showed a trend towards significance for right [F = 3.6; p = 0.06] hippocampal volumes. The significance of this three-way interaction was contributed by presence of ‘diagnosis by genotype’ interaction only in males but not in females. The observed post- hoc power of the study at α = 0.05 for bilateral volumetric differences between diagnostic groups was found to be 87%. However, the observation pertaining how sex modulates the effect of genotype and diagnosis on hippocampal volume should be treated with caution, given the small sample sizes in individual cells [Male GG: GC/CC = 9∶5 (Patients), 14∶6 (Controls); Female GG: GC/CC = 12∶2 (Patients), 13∶4 (Controls)].

**Figure 4:**
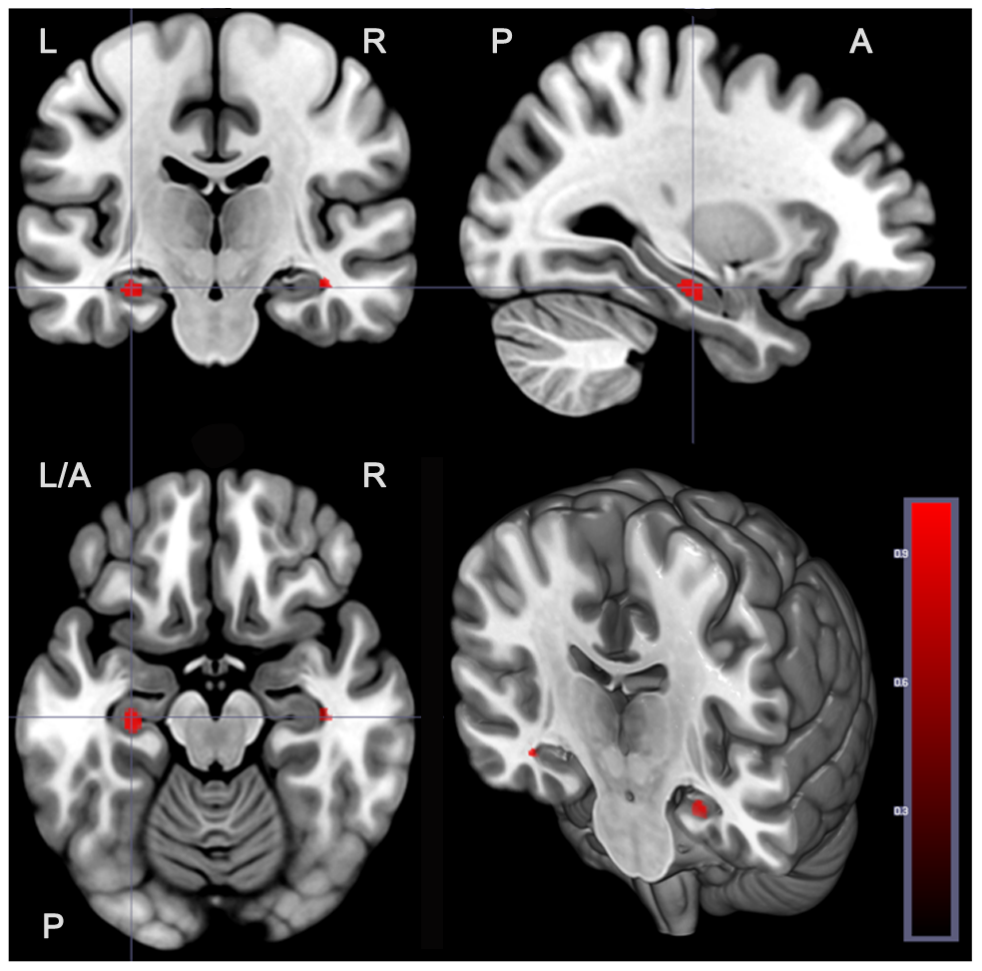
Significant effect of diagnosis-by-genotype interaction on hippocampus volume as demonstrated by voxel based morphometry analysis. Red blobs in the figures show significant diagnosis by genotype interaction involving both right and left hippocampal volumes by VBM in parallel to those that were observed in manual analyses.

In parallel to manual morphometry, automated VBM analysis also showed main effect of diagnosis as well as diagnosis-by-genotype interaction effect on hippocampal gray matter in the same direction (Figure-4). Coordinates, cluster size and peak t value for the VBM analysis of main and interaction effect are presented in Table-3. Further, hippocampal volumes obtained from manual and voxel wise measurements showed a significant and strong positive correlation [Right: r = 0.8; p<0.001; Left: r = 0.8; p<0.001].

**Table 3:** VBM analysis of hippocampal gray matter.

Comparison	Side	MNI Coordinates [X,Y,Z]	Cluster Size	T	df	p
**Main effect of diagnosis**						
**Controls > Patients**	Left	−35 −18 −18	2049	5.13	60	<0.001
	Right	18 −30 −5	2148	5.24	60	<0.001
**Patients > Controls**	Left	Nil	-	-	-	-
	Right	Nil	-	-	-	-
**Diagnosis-by- genotype interaction effect**						
**(GG - controls & GC/CC - patients) > (GC/CC- controls & GG - patients)**						
	Left	−30 −19 −18	61	2.03	58	0.023
	Right	24 −1 −24	37	1.99	58	0.025
	Right	41 −19 −15	29	1.92	58	0.029
**(GC/CC - controls & GG - patients) > (GG - controls & GC/CC - patients)**						
	Left	Nil	-	-	-	-
	Right	Nil	-	-	-	-

**Figure 5:**
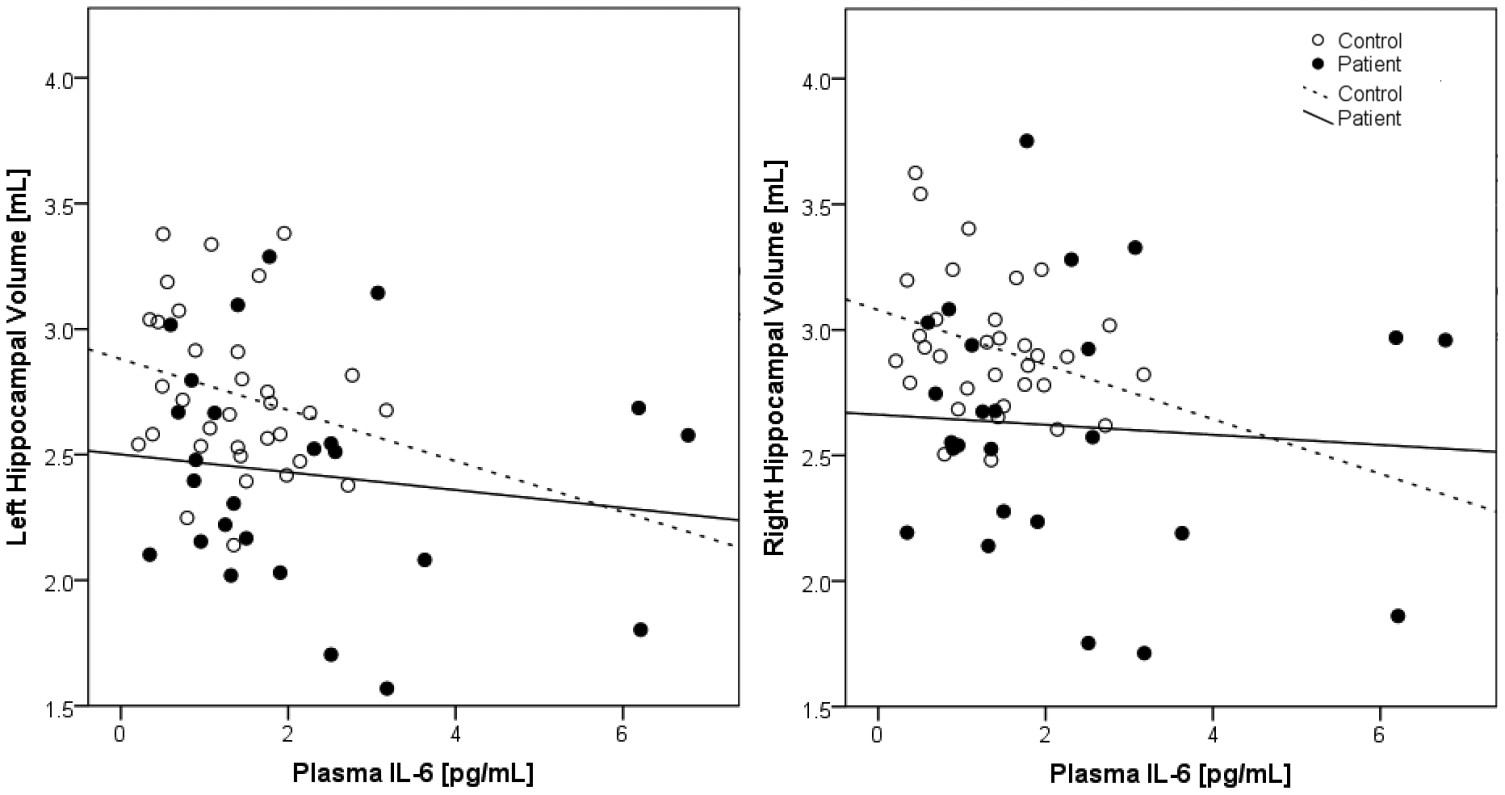
Scatter plot depicting the relationship between plasma IL-6 level and hippocampal volumes in schizophrenia patients and healthy controls.

Plasma IL-6 level had a significant negative correlation with left hippocampal volume (r = −0.3; p = 0.04) and trend-level negative correlation with right hippocampal volume (r = −0.2; p = 0.08) in analyses involving all subjects (patients & controls). Sub-group analyses revealed non-significant negative correlation between IL-6 and bilateral hippocampal volume in controls [Right: r = −0.3; p = 0.09; Left: r = −0.2; p = 0.2] as well as patients [Right: r = −0.1; p = 0.7; Left: r = −0.1; p = 0.5] (Figure-5).

The psychopathology scores of the entire patients sample were: Total SAPS score = 34.9±14.6; Total SANS score = 55.5±26.5. The genotype sub-groups of patients (i.e. GG vs. [GC/CC]) did not significantly differ in total symptoms scores [SAPS: t = 0.9; p = 0.3; SANS: t = 0.5; p = 0.6]. No significant correlations were found between total symptoms scores and IL-6 levels [SAPS: r = 0.2; p = 0.3; SANS: r = 0.2; p = 0.4]. There was a trend towards significant negative correlation between SAPS and hippocampal volume [Right: r = −0.3; p = 0.1; Left: r = −0.4; p = 0.05], but no correlation was found between SANS and hippocampal volume [Right: r = 0.09; p = 0.7; Left: r = 0.2; p = 0.4]

## Discussion

The study observations offer novel insights into the effect of *rs1800795* polymorphism on the hippocampal volume in schizophrenia in comparison with healthy controls. We observed that schizophrenia patients had significantly deficient left and right hippocampal volumes as well as higher plasma IL-6 in comparison with healthy controls. There was a significant diagnosis by genotype interaction involving both right and left hippocampal volumes– i.e. the effect of *rs1800795* genotypes [GG & GG/GC] on hippocampal volume was found to be antithetical between patients and controls. Also, hippocampal volume was significantly deficient only in the subset of patients with “GG” genotype and not with “GG/GC”, in comparison to genotype-matched controls. However, plasma IL-6 showed only weak correlation with hippocampal volumes; there was no significant difference in the frequency distribution of *rs1800795* genotypes between patients and controls; also, IL-6 levels did not show any significant relationship with genotypes.

Various lines of evidence argue for a critical role of hippocampus & its interactions with other brain regions to underlie the pathogenesis of schizophrenia [28], [31], [48], [49], [50]. Interestingly, the influence of hippocampus becomes especially significant given the ‘stress-diathesis’ paradigm as an important explanatory model for schizophrenia [51], [52]. This ‘stress-diathesis’ paradigm invokes the impact of various immunobiological parameters (for example – IL-6 and various other cytokines) [53]. Contextually, our study finding of hippocampal volume deficit is in tune with this proposition.

**Figure 6:**
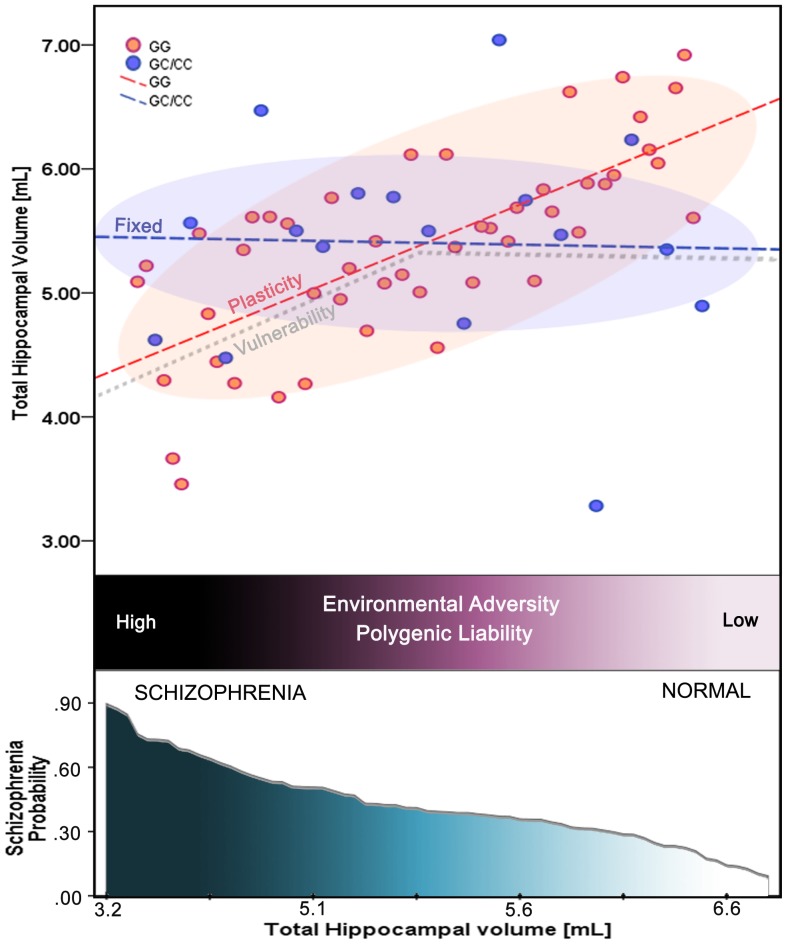
Illustration of the differential susceptibility in contrast to diathesis-stress model. Total hippocampal volume is represented against environmental adversity and polygenic liability that are known to be associated with schizophrenia (not quantified in this study). Pink and blue lines depict homozygous G and carrier of C *rs1800795* genotypes respectively, that differ in their responsiveness to environmental and epistatic factors: the “plasticity” conferred by homozygous G is disproportionately more affected by influences compared to the “fixed” C carrier group. The gray line depicts “vulnerability”, that is affected only when exposed to an adversity, and this diathesis-stress view is not supported by data in the current study. In relation to these models, the bottom panel shows predicted probability of 'schizophrenia' outcome as the function of total hippocampal volume.

In a recent study examining healthy subjects, the polymorphism *rs1800795* showed a strong main effect of genotype with the volume of the right hippocampus head. Homozygous carriers of the G-allele had significantly larger hippocampal gray matter volumes compared to_heterozygous subjects [54]. Our study observation in healthy control group replicates this finding. On the contrary, in patients, the direction of association was reverse – i.e. – those with “GG” genotype had overall smaller hippocampal volume. Thus, these observations pertinent to influence of *rs1800795* promoter polymorphism on the volume of hippocampus are antithetical in controls and patients. Such an antithetical influence which is diametrically differential between patients and controls might support the “differential susceptibility” model (Figure-6). The “differential susceptibility” postulate argues that the same genes that convey increased vulnerability to psychopathology under adverse environmental conditions have the potential to act in advantageous ways on psychological functioning when the environment is supportive or enriched [55], [56]. Interestingly, there is some support that *rs1800795* promoter polymorphism show differential susceptibility in that a study that has applied computational identification of gene–social environment interaction at the human IL-6 locus showed that this polymorphism might have functional impact on gene expression only in the presence of adverse environmental/social conditions [12]. The underlying molecular mechanism has been demonstrated to be mediated through modulation of β-adrenergic activation of GATA1 and particularly GG genotype of *rs1800795* is shown to be GATA1 sensitive. Interestingly, the transcription factor GATA1, is implicated in reduction in brain volume and a decrease in the size and density of neurons [57]. Thus, it is possible that under the influence of environmental factors, the GG polymorphism might have resulted in deleterious impact on neurodevelopment leading to decreased hippocampal volume.

Hippocampus is among the brain regions with the most pronounced expression of IL-6 receptors [58] and is sensitive to IL-6 in the peripheral blood, which can enhance dopamine turnover in the hippocampus [59] . Animal studies of prenatal immune activation have shown that maternal IL-6 can cross the placenta [60] and disrupt fetal brain development [61] by potentially inhibiting neuronal survival [62], dendritic outgrowth and branching of hippocampal and cortical neurons [63]. Further, fetal exposure to IL-6 in rats can lead to increased IL-6 levels in the peripheral circulation and hippocampus as well as abnormalities of hippocampal structure, morphology and function during adulthood [64].

On the other hand, in absence of inflammatory response, IL-6 can act as neuropoietic cytokine and neurotrophic agent [65], plays critical role in neurite outgrowth, survival, proliferation and gene expression [66]. Further, pathological increase or complete deficiency of IL-6 both can result in impairment of hippocampal dependent function like learning in mice [67] implicating that perhaps an appropriate IL-6 balance might be crucial for normal neurodevelopment and function.

We observed that, in the subgroup with “GC/CC” genotype, the hippocampal volumes were not significantly deficient in patients in comparison to genotype matched controls. Perhaps, this could be seen as neuroprotective effect of “C” allele in schizophrenia patients. However, this effect was seen only in male patients. In this context, it is noteworthy that exposure to sex hormones like androgen and estrogen potentially modulates IL-6 production [68], [69]. Moreover, genetic predisposition to produce high levels of IL-6 by *rs1800795* could itself be sex-dependent with the earlier study reporting the effect of this SNP on serum IL-6 in men, but not in women [70]. Further, another study has shown sex differences in terms of hippocampal sub regions which were affected in rats prenatally exposed to IL-6 [64]. However, it is important to note that the sample size of this study is very small given the difficulty in recruiting antipsychotic-naïve schizophrenia patients for these studies. Hence, the potential modulatory influences of the sex on the effect of genotype and diagnosis on hippocampal volume should be treated with caution, given the small sample sizes in individual cells.

The image analysis methodologies used in this study merit further discussion. While manual morphometry reliably informs about overall volume differences over a specified region, VBM provides more detail regarding the specific points of maximal change within a structure. Both of these methods have been demonstrated to be capable of detecting differences in small areas such as the hippocampus [71], [72], [73], [74], [75]. Particularly, VBM is free of rater and anatomic variability related reliability issues and biases [76]. However, VBM is known to be sensitive to optimization methods (e.g. creation of study-specific template), smoothing parameters (FWHM of Gaussian kernel) [77] and may be prone to errors such as mis-registration of small regions of interest and tissue misclassification [78].

On the contrary, manual volumetric techniques are prone for rater-dependent errors. To minimize such errors, in this study, we have ascertained optimal inter-rater reliability. Moreover, the rater who performed the volumetry was blind to the status of the subject since the analysis was performed on coded images. In addition, it is important to note that hippocampal volumes reported in our study range from 2.4 to 3.6 ml (right) and 2.1 to 3.4 ml (left) for healthy controls, 1.7 to 3.8 ml (right) and 1.6 to 3.3 ml (left) for patients [Mean ± SD given in Table-2]; this is comparable with various earlier studies that have examined hippocampal volume of schizophrenia patients and normal individuals [79], [80], [81], [82]. Nonetheless, it is noteworthy that a large discrepancy in mean hippocampal volume can be seen in available literature, due to factors such as heterogeneous procedures for anatomic definition of the hippocampus, different approaches to measure volume, i.e. manual vs. automated methods, inclusion of white and gray matter in the definition of the region etc. Data from online database - ‘The Internet Brain Volume Database’ (http://www.cma.mgh.harvard.edu/ibvd) shows that average unadjusted hippocampal volumes among normal individuals in the age group similar to that of our study (17–42 yr.), from 67 studies published from 2000–2010 are 3.3 ml for left (ranging from 2.1 to 4.5 ml) and 3.4 ml for right (ranging from 2.1 to 4.7 ml). We find that hippocampal volumes of healthy controls in_ our study approximately correspond to the first quartile of these reported values (left − 2.8 ml, right – 3.0 ml). To summarize, the hippocampal volumes obtained in this study is comparable with many previous studies in this area. It has been suggested earlier [83] that application of a combination of both of these methods (manual as well as VBM) might provide highest reliability and accuracy. Hence, combined application of these image analysis methods has added to the methodological strength of this study.

In this study, we have observed a significant impact of IL-6 genotype on bilateral hippocampus in healthy controls with the directionality of effect in tune with a previous observation [54]; on the other hand, in schizophrenia patients the effect of G-allele on the hippocampal volume was restricted to the right side. With regards to the lateralization of hippocampus volume deficits in schizophrenia, while few studies have shown significant volume deficits in bilateral hippocampi in antipsychotic-naive schizophrenia [84], [85], others have shown deficits to be significantly pronounced in one of the hemisphere, either right [86], [87] or left [88]. Keeping this in mind, we have examined right and left hemisphere separately. An important future direction would be to explore for any consistent observation to support status-differential genotypic effects with regards to the laterality of hippocampal involvement in larger samples.

While our study observations have implicated global hippocampal deficits to be linked IL-6 promoter polymorphism, one potential area of further enquiry would be the specific relationship of sub-regions of hippocampus in terms of any differential associations. We used 3Tesla MR images with 1 mm resolution, which allows efficient segmentation of hippocampus in manual morphometry as well as VBM. However, the methods used in this study do not permit the accurate localization of maximal volume change within a relatively small brain structure such as hippocampus. We believe that this is an important aspect that needs to be evaluated in future studies. Also, it is noteworthy that the correlation between hippocampus and IL-6 in the whole sample seems weak despite the observations that the hippocampi are smaller in patients with concurrent greater levels of plasma IL-6. Perhaps, examination of a larger sample might facilitate further elucidation of this relationship. This is another important future direction.

In summary, our first time observations suggest significant relationship between GG allele of IL6 promoter polymorphism *rs1800795* and reduced hippocampal volume in antipsychotic-naïve schizophrenia patients. Moreover, this relationship was antithetical in healthy controls, and this effect was observed in men but not in women. Together, these observations support a “differential susceptibility” effect of *rs1800795* in schizophrenia pathogenesis mediated through hippocampal volume deficit that is of possible neurodevelopmental origin.

## Supporting Information

Material S1 Comparative analysis of one-to-on matched samples of patients and controls.
(DOCX)
