# Supplementary material for: Relationship between Interleukin-6 Gene Polymorphism and Hippocampal Volume in Antipsychotic-Naïve Schizophrenia: Evidence for Differential Susceptibility?
Source: PLoS One. 2014 May 2;9(5):e96021. doi: 10.1371/journal.pone.0096021 (PMC4008499; doi:10.1371/journal.pone.0096021)
Supplement: Material S1 — Comparative analysis of one-to-on matched samples of patients and controls. (DOCX) [file pone.0096021.s001.docx]

**Supplementary Material - S1**

**Comparative analysis of one-to-on matched samples of patients and controls**

As the control group consisted of a larger number of subjects than the patients’ group, it is possible that this might have differentially influenced the variance of the measurements between groups. To explore this further, we performed additional analyses involving patients (N=28) and equal number of controls (N=28) with one-to-one matching. The results of these analyses did not differ from the observations from the whole group

**Comparative profile of schizophrenia patients and one-to-one matched healthy controls**

| **Characteristic** | **Patients** | **Controls** | **Statistic** | **p** |
| --- | --- | --- | --- | --- |
| **N** | **28** | **28** |  |  |
|  |  |  |  |  |
| Age [Years] * | 29.9±5.7 | 28.1±5.3 | t=1.2 | 0.2 |
| Sex Ratio [M:F] ^$^ | 14:14 | 14:14 | χ^2^=0 | 1.0 |
| Left Hippocampal Volume [mL]^#^ | 2.4±0.4 | 2.7±0.3 | F=7.2 | 0.01 |
| Right Hippocampal Volume [mL]^#^ | 2.6±0.5 | 2.9±0.3 | F=7.2 | 0.01 |
| Plasma IL-6 [pg/mL] *^,£^ | 2.2±1.8 | 1.4±0.8 | t=2.2 | 0.03 |
| *IL-6* Genotype [GG : GC/CC]^$^ | 21:7 | 21:7 | χ^2^=0 | 1.0 |

* - Independent samples t-test; ^$^ - Chi-Square test; ^#^ - ANCOVA

£ - data was available for 25 patients and 25 controls

Multivariate ANCOVA on bilateral hippocampal volume, controlling for the potential confounding effects of age, sex and total brain volume revealed a significant ‘diagnosis by genotype’ interaction [F=5.7; p=0.006] involving both right [F=11.6; p=0.001] and left [F=8.2; p=0.006] hippocampal volumes (Figures below). Follow-up analyses to uncover the effect of genotype identified that in the subgroup of subjects who carried “GG” genotype, the hippocampal volumes were significantly deficient in patients in comparison with healthy controls [Right: F=20.0; p<0.001; Left: F=19.4; p<0.001], whereas in the subgroup with “GC/CC” genotype, the hippocampal volumes did not differ significantly [Right: F=1.1; p=0.3; Left: F=1.0; p=0.3]. Within healthy controls, GG homozygotes had significantly larger bilateral hippocampal volume compared to GC/CC carriers [Right: F=5.3; p=0.03; Left: F=5.0; p=0.04]; on the contrary, the reverse was true in patients [but significant only for right side; Right: F=5.5; p=0.03; Left: F=1.7; p=0.2].


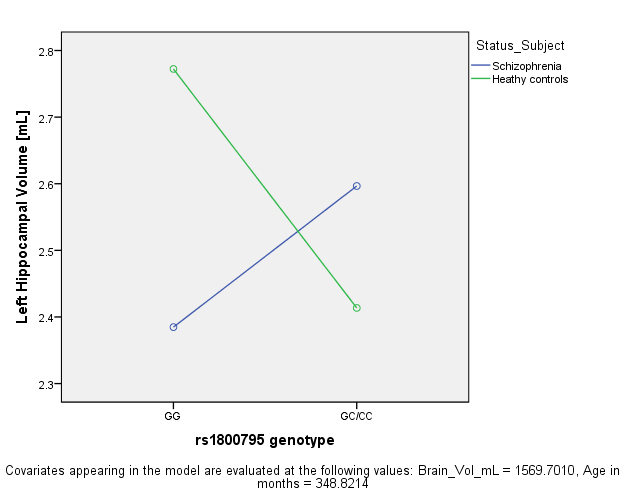


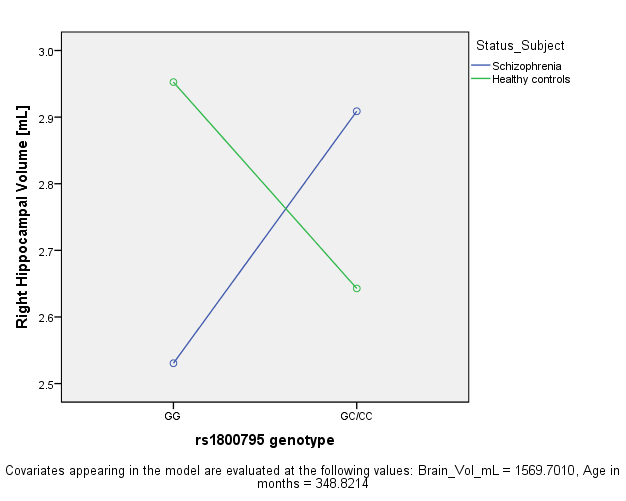


---------------------------------------------------------------------------------------------------------------------------------------
